# Aberrant expression of OATP1B3 in colorectal cancer liver metastases and its clinical implication on gadoxetic acid-enhanced MRI

**DOI:** 10.18632/oncotarget.20295

**Published:** 2017-08-16

**Authors:** Seung Hyun Park, Honsoul Kim, Eun Kyung Kim, Hogeun Kim, Dong Kyu Choi, Yong Eun Chung, Myeong-Jin Kim, Jin-Young Choi

**Affiliations:** ^1^ Department of Radiology and Research Institute of Radiological Science, Severance Hospital, Yonsei University College of Medicine, Seoul, Korea; ^2^ Department of Pathology, Severance Hospital, Yonsei University College of Medicine, Seoul, Korea; ^3^ New Drug Development Center, Daegu-Gyeongbuk Medical Innovation Foundation, Daegu, Korea

**Keywords:** colorectal cancer (CRC), liver metastases (LM), magnetic resonance imaging (MRI), gadoxetic acid (Gd-EOB-DTPA), organic anionic transporting polypeptide (OATP)

## Abstract

**Purpose:**

To investigate the factors associated with hepatobiliary phase (HBP) enhancement at gadoxetic acid-enhanced magnetic resonance imaging (MRI) and to determine whether HBP images could be used to predict prognosis in patients with colorectal cancer liver metastasis (CRLM).

**Results:**

Of the 96 total nodules, 65 and 31 nodules were in the mixed and clearly hypointense groups, respectively. In the 55 nodules without preoperative chemotherapy, organic anionic transporting polypeptide 1B3 (OATP1B3) expression was a significant factor regarding the HBP enhancement (*P*=0.042). In this subgroup, nodules with OATP1B3 expression displayed a significantly higher relative intensity ratio on the HBP image (RIR_post_) and relative enhancement ratio (RER) than those lacking this marker (*P*=0.024, 0.003, respectively). No significant factor was associated with the enhancement pattern in the chemotherapy group. The mixed hypointense group displayed worse survival rates (*P*=0.002).

**Materials and Methods:**

Ninety-six patients who underwent pre-operative liver MRI and surgical resection for CRLM from January 2010 to June 2012 were retrospectively analyzed. We qualitatively evaluated the HBP enhancement pattern of CRLMs and classified them into mixed and clearly hypointense groups. For quantitative measurement, the RIR_post_ and RER were analyzed. To investigate factors associated with HBP enhancement, tumor components (fibrosis, necrosis, and cellularity) and OATP1B3 expression were scored on a 4-point scale. Univariate and multivariate analyses were done to determine significant factors for visual enhancement and quantitative parameters.

**Conclusions:**

OATP1B3 expression is associated with mixed hypointense CRLMs without chemotherapy. Signal intensity on HBP has potential usefulness to predict prognosis in CRLMs.

## INTRODUCTION

Colorectal cancer (CRC) is the third-most common cancer worldwide [1, 2]. Hepatic metastases from CRC are an important clinical problem because the liver is the most frequent site of metastasis, and their presence can significantly change the treatment plan and outcome [3]. Gadoxetic acid-enhanced MRI is an established method widely used for the evaluation of colorectal cancer liver metastasis (CRLM) because the hepatobiliary phase (HBP) offers excellent lesion to liver contrast, leading to a higher lesion detection rate [4].

Gadoxetic acid is a liver-specific MRI contrast agent whose uptake is mediated by the organic anionic transporting polypeptide 1B3 (OATP1B3) expressed by hepatocytes. The general belief has been that tumors of non-hepatocyte origin, including CRLMs, could not actively uptake gadoxetic acid and, therefore, lacked a T1 contrast effect on the HBP [5]. However, a recent study reported that 72% of CRLMs show mixed signal intensity (SI) on HBP, vaguely assumed to be the result of gadoxetic acid retention within the extracellular space where abundant desmoplasia and intraacinar necrosis exist [6], but the underlying mechanism remains poorly understood.

Accumulating evidence indicates that OATP1B3 is, in fact, not exclusively expressed by hepatocytes; aberrant OATP expression occurs in various human malignancies arising from the colon, pancreas, gall bladder, lung, and breast in addition to hepatocellular carcinomas [7–14]. Moreover, experimental evidence has indicated that aberrant expression of OATP1B3 is associated with decreased apoptosis following chemotherapy and more aggressive tumor behavior, implicating the expression of this marker as a potentially adverse prognostic factor [13]. However, to our knowledge no study has been performed to elucidate whether or not such aberrant expression of OATP1B3 has an influence on the imaging features of a gadoxetic acid-enhanced MRI.

We hypothesized that if OATP1B3 is responsible for the intracellular transport of gadoxetic acid, theoretically the aberrant expression of OATP1B3 may influence the MRI enhancement pattern even in tumors of non-hepatocyte origin.

The purpose of this study was to elucidate the factors that might influence the enhancement pattern of a CRLM on a gadoxetic acid-enhanced MRI and, if so, whether they were associated with the clinical outcome.

## RESULTS

### Enhancement pattern of the metastatic liver nodules

**Figure 1:**
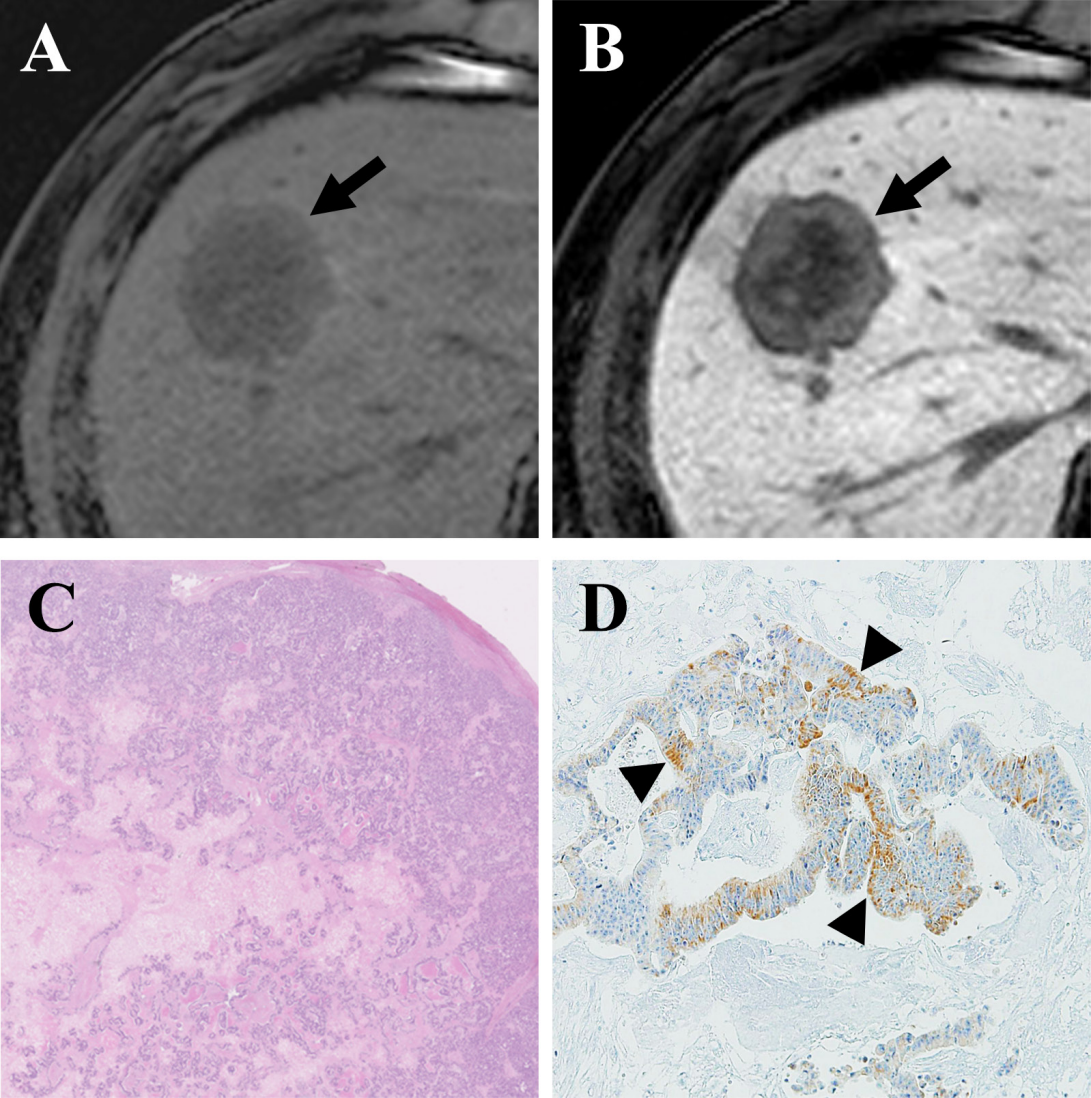
Gadoxetic acid-enhanced MRI of a CRLM diagnosed by surgical resection in a 61-year-old man **(A)** Precontrast T1-weighted image showed a hypointense nodule (arrow) in segment 8. **(B)** The tumor (arrow) showed mixed hypointensity during the hepatobiliary phase (HBP) of a liver dynamic MRI. **(C)** Hematoxylin and eosin staining revealed grade 1 fibrosis, grade 2 central necrosis, and grade 3 cellularity. **(D)** Immunohistochemistry (×100) revealed weak expression of OATP1B3 (arrowhead) in 20% of the tumor.

**Figure 2:**
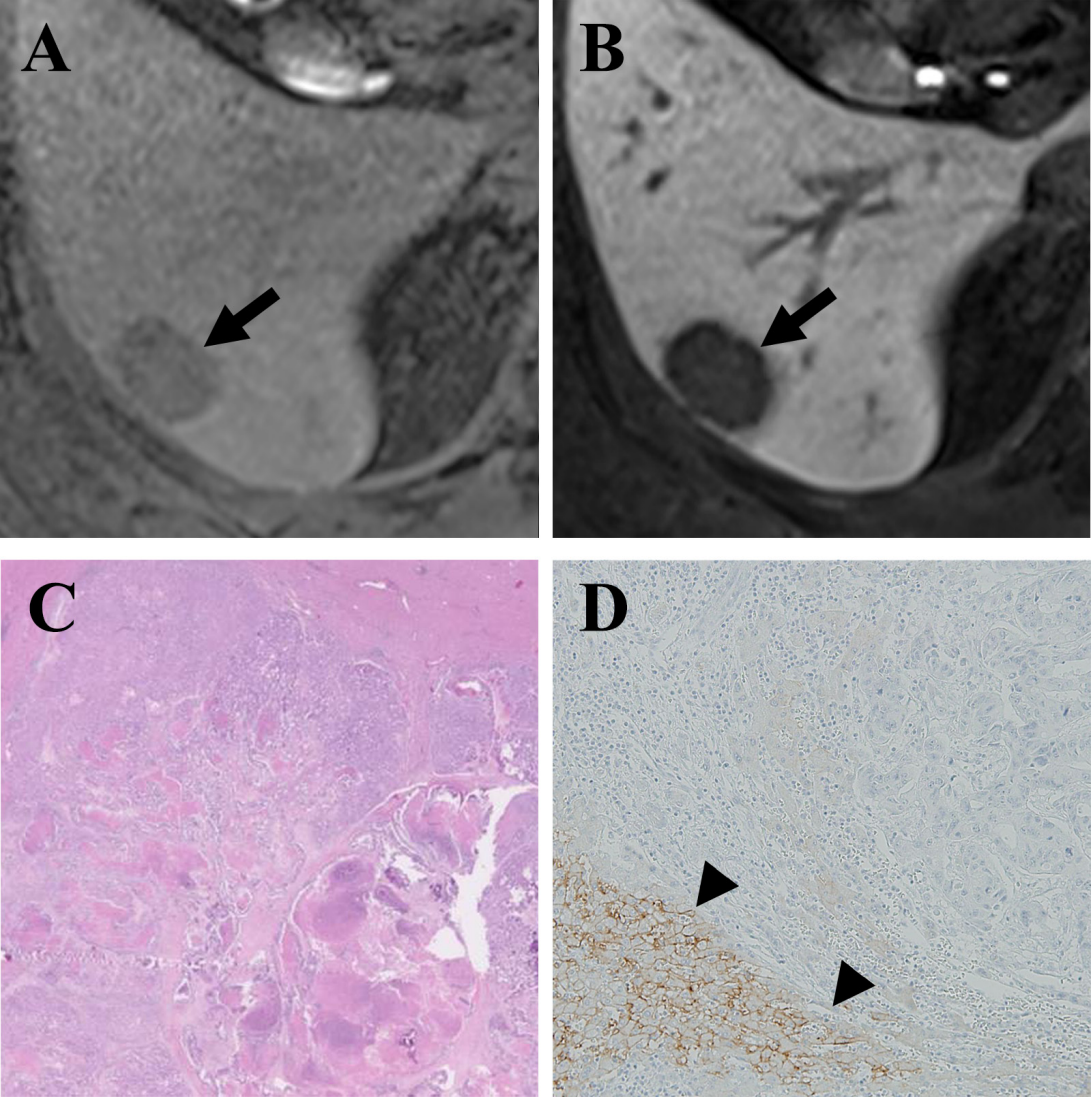
Gadoxetic acid-enhanced MRI of a CRLM diagnosed by surgical resection in a 49-year-old man **(A)** Precontrast T1-weighted image showed a hypointense nodule (arrow) in segment 6. **(B)** The tumor (arrow) showed a clear hypointensity during the hepatobiliary phase (HBP) of a liver MRI. **(C)** Hematoxylin and eosin staining revealed grade 1 fibrosis, grade 2 central necrosis, and grade 3 cellularity. **(D)** Immunohistochemistry (×100) revealed a lack of expression of OATP1B3 in the tumor but strong expression in the surrounding liver parenchyma (arrowhead).

Among the 96 nodules (Supplementary Figure 1), 65 nodules (68%) and 31 nodules (32%) were classified as mixed hypointense nodules and clearly hypointense nodules, respectively (Figures 1–2). Among the patients who did not receive preoperative chemotherapy (non-chemotherapy group: n=55), 34 nodules (62%) showed a mixed hypointense signal intensity (SI), and 21 nodules (38%) showed a clearly hypointense SI in the HBP. In the chemotherapy group (n=41), 31 nodules (76%) showed a mixed hypointense SI, and 10 nodules (24%) showed a clearly hypointense SI. There was no significant difference in the enhancement pattern between the non-chemotherapy and chemotherapy groups (*P*=0.1529).

### Histopathologic parameters and OATP1B3 immunohistochemistry

The histopathological parameters were analyzed as fibrosis grade (G1, 59 [61%]; G2, 25 [26%]; G3, 9 [9%]; and G4, 3 [3%]), necrosis grade (G1, 32 [33%]; G2, 23 [24%]; G3, 19 [20%]; and G4, 22 [23%]), and cellularity grade (G1, 36 [38%]; G2, 19 [20%]; G3, 28 [29%]; and G4, 13 [14%]), respectively. The results of each parameter within the chemotherapy group and the non-chemotherapy group were also provided (Table 1).

**Table 1:** Result of univariate and multivariate analyses of histopathologic factors accounting for the visual enhancement pattern

	Mixed hypointense	Clearly hypointense	Univariate analysis		Multivariate analysis		
			Exp (B) value	P value	Exp (B) value	95% confidence interval	P value
Non preoperative chemotherapy group							
OATP1B3 (-/+)	19/15 (56%/44%)	16/5 (76%/24%)	2.526	0.134	5.694	1.067–30.379	**0.042**
Fibrosis (G1/G2-4)	28/6 (82%/18%)	13/8 (62%/38%)	0.348	0.097	0.197	0.046–0.850	**0.029**
Necrosis (G1/G2-4)	14/20 (41%/59%)	7/14 (33%/67%)	0.714	0.561	1.165	0.274–4.960	0.837
Cellularity (G1/G2-4)	11/23 (32%/68%)	5/16 (24%/76%)	1.530	0.499	3.301	0.737–14.792	0.119
Preoperative chemotherapy group							
OATP1B3 (-/+)	19/12 (61%/39%)	6/4 (60%/40%)	1.056	0.942	1.025	0.227–4.635	0.975
Fibrosis (G1/G2-4)	12/19 (39%/61%)	6/4 (60%/40%)	0.421	0.245	2.413	0.501–11.631	0.272
Necrosis (G1/G2-4)	9/22 (29%/71%)	2/8 (20%/80%)	1.636	0.577	0.754	0.116–4.911	0.768
Cellularity (G1/G2-4)	16/15 (52%/48%)	4/6 (40%/60%)	1.600	0.525	1.880	0.416–8.490	0.412

* Exp(B): Exponentiation of the B coefficient, OATP1B3: Organic anionic transporting polypeptide 1B3.

OATP1B3 immunohistochemistry revealed 30 nodules showing weakly positive expression and 6 nodules with moderately positive expression of OATP1B3. In concordance with a previous report [13], we observed that unlike normal liver tissue (positive control, Supplementary Figure 2) that displayed a membranous OATP1B3 expression, all of these OATP1B3-positive CRLM nodules (n=36) showed cytoplasmic OATP1B3 expression. The other 60 nodules lacked OATP1B3 activity.

### Correlation of the qualitative imaging analysis with the histopathology

**Figure 3:**
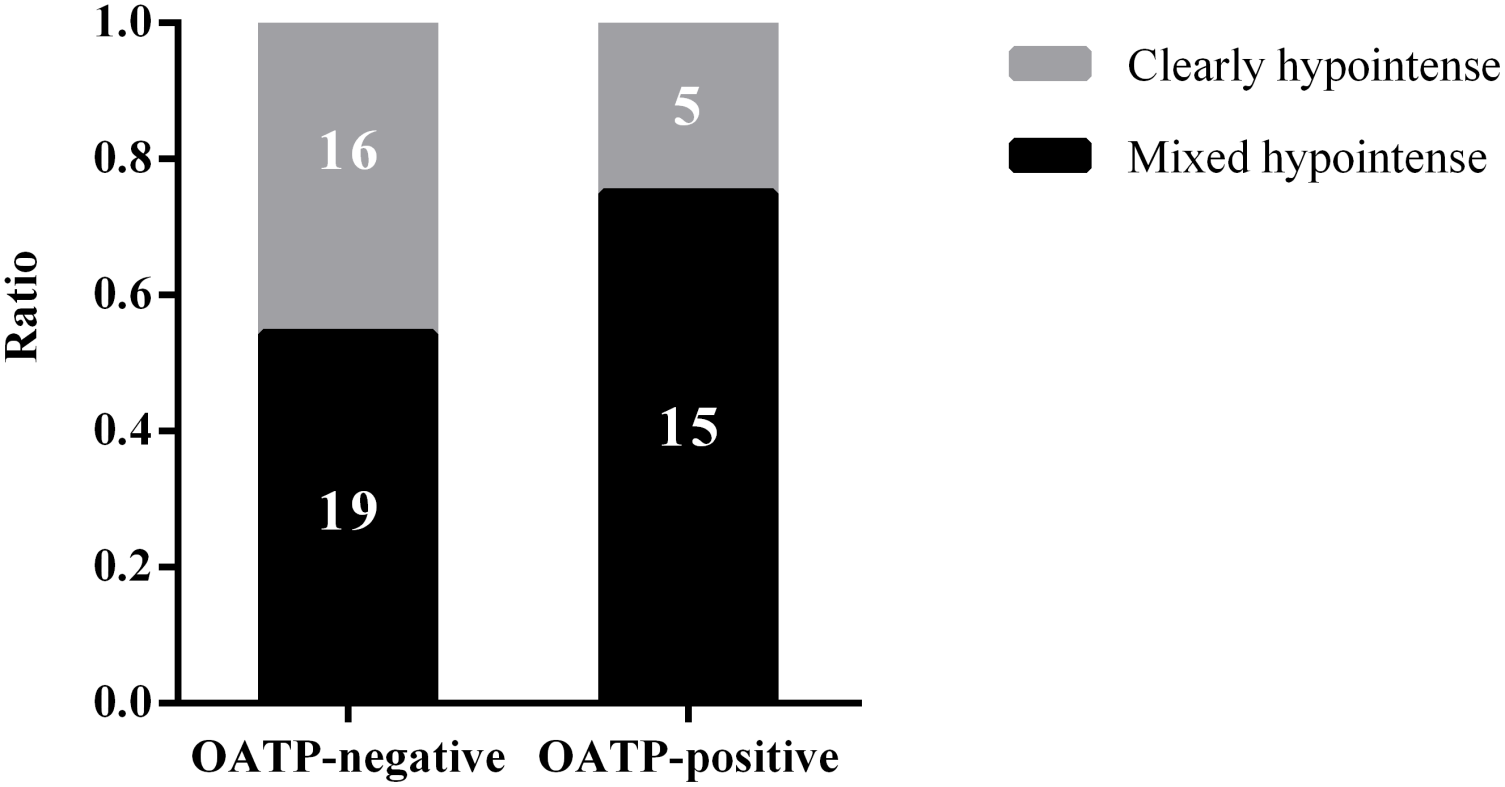
A graph showing the proportion of clearly hypointense and mixed hypointense CRLMs without chemotherapy in relation to OATP1B3 expression

We separately performed univariate and multivariate analyses for the chemotherapy and non-chemotherapy groups. In the non-chemotherapy group (n=55), no factor seemed to be significantly associated with the enhancement pattern of CRLMs in univariate analysis. However, the multivariate analysis identified positive OATP1B3 expression (*P*=0.042) and fibrosis grade (*P*=0.029) as factors significantly associated with the HBP enhancement pattern (Table 1). In specific, 19 of the 35 OATP1B3-negative CRLM nodules (54%) displayed a mixed hypointense pattern during the HBP; whereas, 15 of the 20 OATP1B3-positive CRLM cases (75%) were mixed hypointense nodules (Figure 3). In the non-chemotherapy group, fibrosis was also significant factor showing an inverse correlation with the enhancement pattern (*P*=0.029; Table 1). In the chemotherapy group, we could find no significant factor associated with the enhancement pattern in both univariate and multivariate analyses.

### Correlation of the quantitative imaging analysis with the histopathology

**Figure 4:**
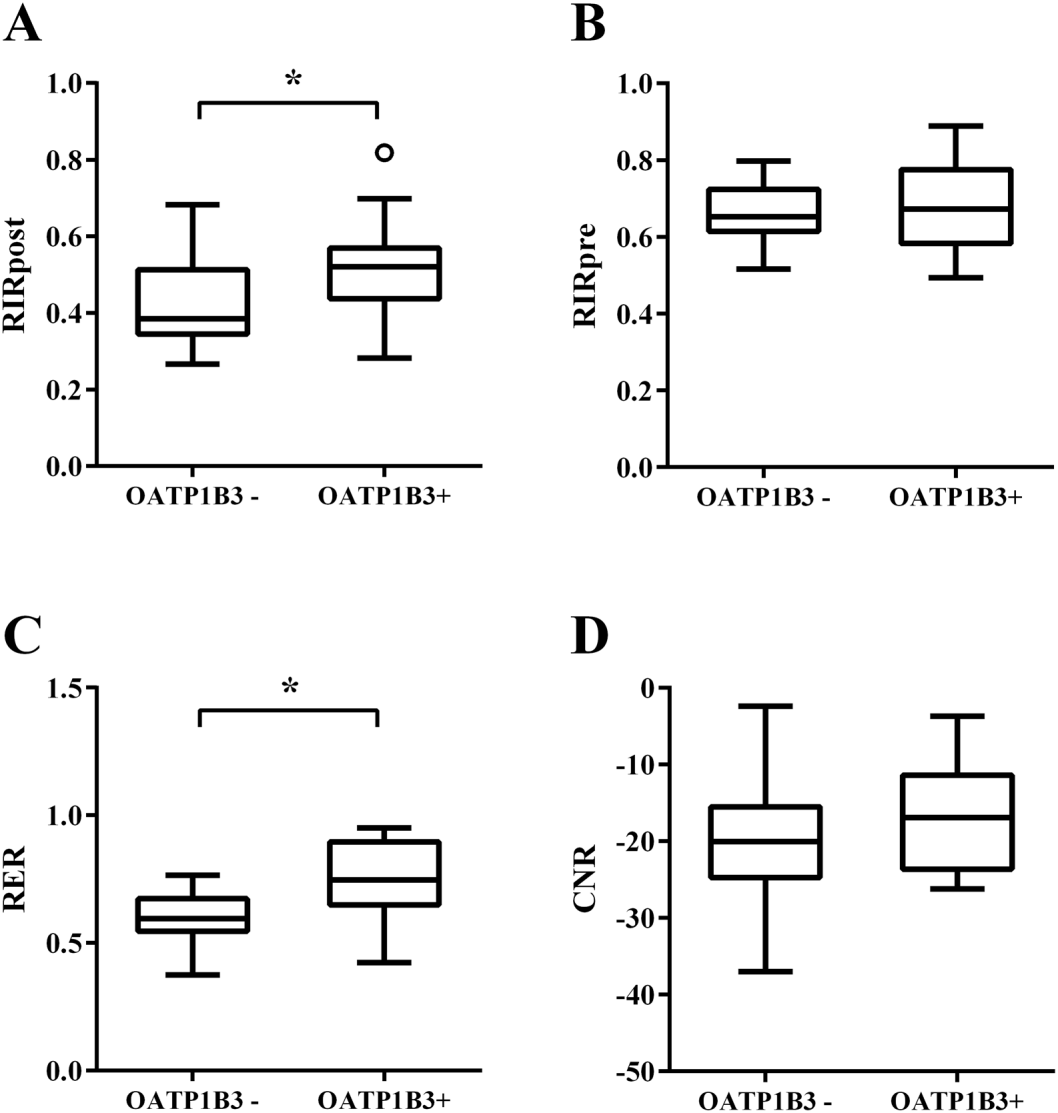
Box plots showing four MR quantitative parameters of CRLMs without chemotherapy **(A)** relative intensity ratio on the hepatobiliary phase (HBP) image (RIR_post_), **(B)** relative intensity ratio on the precontrast image (RIR_pre_), **(C)** relative enhancement ratio (RER), and **(D)** contrast to noise ratio (CNR). * P value < 0.05.

In the non-chemotherapy group, the mean relative intensity ratio on the HBP image (RIR_post_), relative intensity ratio on the precontrast image (RIR_pre_), relative enhancement ratio (RER), and contrast to noise ratio (CNR) values were 0.46, 0.68, 0.67, and -19.62, respectively. In the univariate analysis, nodules positive for OATP1B3 expression possessed significantly higher RIR_post_ and RER values when compared with that of the lesions without OATP1B3 expression (*P*=0.014, 0.005, respectively; Table 2 and Figure 4). Nodules with high-grade cellularity (G2-4) also possessed significantly higher CNR values than that of the nodules with low-grade (G1) cellularity (*P*=0.029; Table 2). In the multivariate analysis, positive OATP1B3 expression was associated with higher RIR_post_ and RER values in comparison with lesions lacking OATP1B3 expression (*P*=0.024, 0.003, respectively; Table 3 and Figure 4). However, cellularity failed to reach statistical significance in the multivariate analysis, including CNR (*P*=0.144).

**Table 2:** Results of the univariate analyses of histopatholgic factors for the quantitative parameters

	RIR_post_	RIR_pre_	RER	CNR	Univariate analysis							
					RIR_post_		RIR_pre_		RER		CNR	
					B value	P value	B value	P value	B value	P value	B value	P value
Non preoperative chemotherapy group												
OATP1B3 (-/+)	0.4244/0.5109	0.6780/0.6836	0.6274/0.7467	-21.1087/-17.0274	0.086	**0.014**	0.006	0.860	0.119	**0.005**	4.081	0.074
Fibrosis (G1/G2-4)	0.4632/0.4345	0.6806/0.6785	0.6820/0.6378	-19.0491/-21.3100	-0.029	0.472	-0.002	0.954	-0.044	0.364	-2.261	0.375
Necrosis (G1/G2-4)	0.4927/0.4331	0.6963/0.6700	0.7012/0.6520	-19.7470/-19.5489	-0.060	0.091	-0.026	0.399	-0.049	0.258	0.198	0.931
Cellularity (G1/G2-4)	0.4560/0.4558	0.6782/0.6808	0.6740/0.6695	-23.3485/-18.0968	<0.001	0.996	0.003	0.937	-0.005	0.923	5.252	**0.029**
Preoperative chemotherapy group												
OATP1B3 (-/+)	0.5152/0.4902	0.7597/0.7097	0.6791/.6970	-16.2539/-16.9190	-0.025	0.540	-0.050	0.199	0.018	0.690	-0.665	0.771
Fibrosis (G1/G2-4)	0.4851/0.5213	0.7381/0.7414	0.6705/0.6986	-17.2433/-15.9417	0.036	0.364	0.003	0.931	0.028	0.524	1.302	0.562
Necrosis (G1/G2-4)	0.5572/0.4875	0.7499/0.7365	0.7440/0.6663	-14.7342/-17.1296	-0.070	0.121	-0.013	0.760	-0.078	0.117	-2.395	0.347
Cellularity (G1/G2-4)	0.5156/0.4945	0.7604/0.7185	0.6792/0.6935	-15.7042/-17.3682	-0.021	0.596	-0.042	0.273	0.014	0.744	-1.664	0.455

* RIR_post_: Relative intensity ratio on the HBP image, RIR_pre_: Relative intensity ratio on the precontrast image, RER: Relative enhancement ratio, CNR: Contrast to noise ratio, OATP1B3: Organic anionic transporting polypeptide 1B3.

**Table 3:** Results of the multivariate analyses of histopathologic factors for the quantitative parameters

	Multivariate analysis											
	RIR_post_			RIR_pre_			RER			CNR		
	B value	95% CI	P value	B value	95% CI	P value	B value	95% CI	P value	B value	95% CI	P value
Non preoperative chemotherapy group												
OATP1B3 (-/+)	0.093	0.013–0.174	**0.024**	-0.008	-0.084–0.174	0.841	0.150	0.054–0.174	**0.003**	3.985	-1.276–0.174	0.134
Fibrosis (G1/G2-4)	-0.053	-0.131–0.024	0.171	-0.005	-0.078–0.024	0.892	-0.077	-0.169–0.024	0.101	-2.410	-7.435–0.024	0.340
Necrosis (G1/G2-4)	-0.028	-0.131–0.047	0.455	-0.030	-0.101–0.047	0.400	0.002	0.087–0.04	0.956	1.569	-3.318–0.047	0.522
Cellularity (G1/G2-4)	-0.036	-0.114–0.041	0.352	0.005	-0.069–0.041	0.901	-0.061	-0.154–0.041	0.190	3.729	-1.320–0.041	0.144
Preoperative chemotherapy group												
OATP1B3 (-/+)	-0.021	-0.103–0.062	0.611	-0.051	-0.131–0.029	0.201	0.022	-0.070–0.114	0.629	-0.526	-5.234–4.182	0.822
Fibrosis (G1/G2-4)	0.019	-0.067–0.104	0.658	-0.004	-0.087–0.078	0.919	0.011	-0.084–0.106	0.819	0.808	-4.070–5.686	0.739
Necrosis (G1/G2-4)	-0.069	-0.166–0.028	0.157	-0.022	-0.116–0.072	0.639	-0.074	-0.183–0.034	0.172	-2.519	-8.064–3.026	0.363
Cellularity (G1/G2-4)	-0.034	-0.116–0.047	0.396	-0.046	-0.125–0.032	0.242	0.002	-0.088–0.093	0.957	-2.157	-6.797–2.483	0.353

* RIR_post_: Relative intensity ratio on the HBP image, RIR_pre_: Relative intensity ratio on the precontrast image, RER: Relative enhancement ratio, CNR: Contrast to noise ratio, CI: confidence interval, OATP1B3: Organic anionic transporting polypeptide 1B 3.

In the chemotherapy group, the mean RIR_post_, RIR_pre_, RER, and CNR values were 0.51, 0.74, 0.69, and -16.52, respectively. None of the parameters we examined reached statistical significance in regard to the quantitative parameters (RIR_post_, RIR_pre_, RER, and CNR values) in either the univariate or multivariate analyses (Tables 2–3).

### Imaging features and clinical outcome

The mean follow-up interval for the 96 patients was 1336 days (range 59–2555 days). Forty-eight patients died during the follow-up period. Sixty-four patients developed local recurrence of primary colon cancer and/or metastases after a curative surgical resection; local recurrence of the primary colon cancer at the resection site occurred in two patients. Metachronous distant metastases developed in the liver (n=12), liver and lung (n=11), liver and peritoneum/omentum (n=6), liver and lymph node (n=2), liver and brain (n=1), lung (n=15), lung and peritoneum/omentum (n=1), lung and lymph node (n=1), lung and ovary (n=1), lymph node (n=8), peritoneum/omentum (n=3), and brain and spinal cord (n=1).

**Figure 5:**
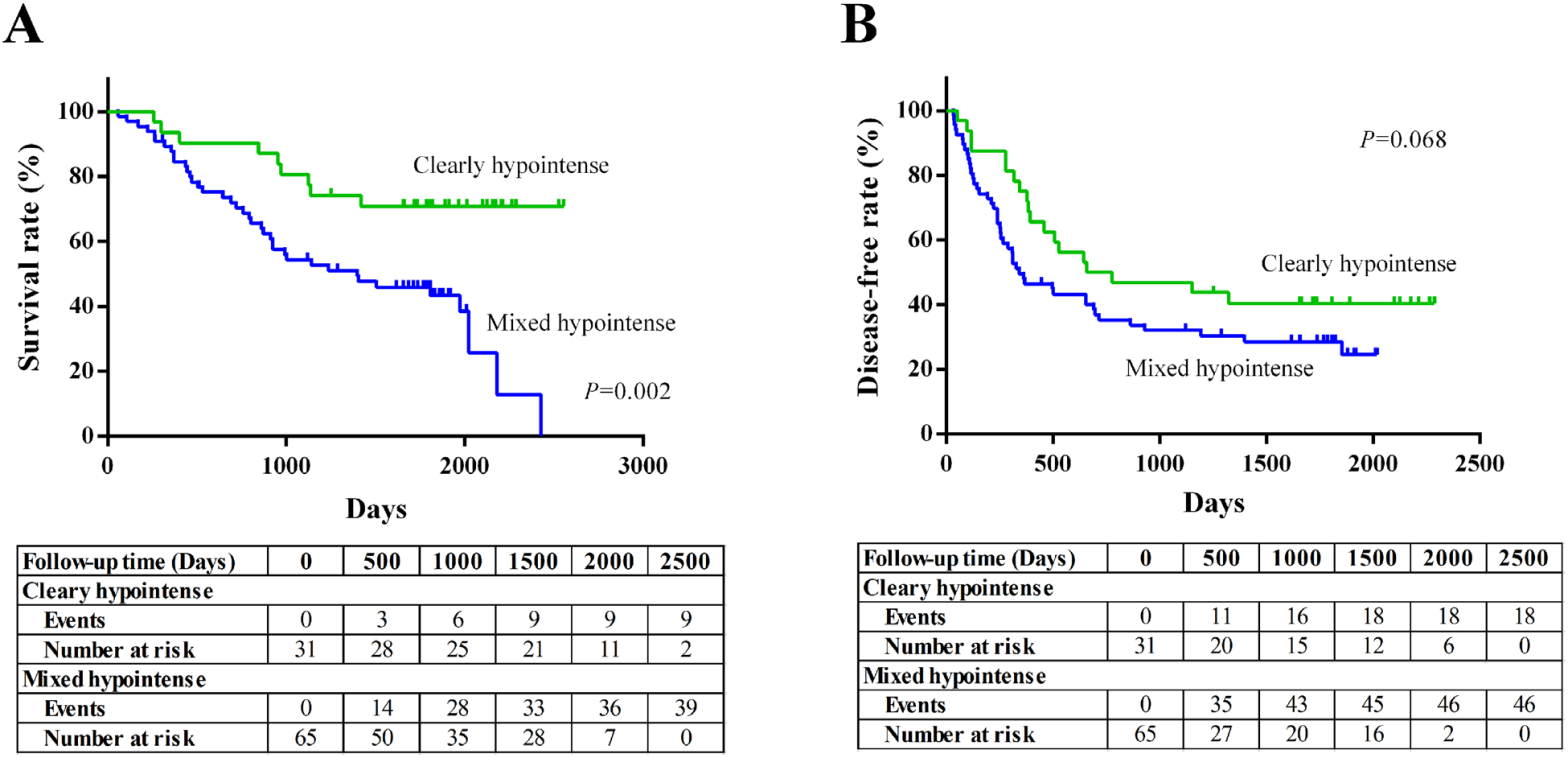
Kaplan-Meier survival curves in CRLM patients based on the visual enhancement pattern of the tumor during the hepatobiliary phase (HBP)

The estimated 5-year survival rate was 52.5%, and the mean survival time was 1623 days. The estimated 5-year disease-free survival rate was 33.1%, and the mean recurrence-free time was 1004 days. The patients with mixed hypointense nodule(s) showed inferior overall survival when compared with those of patients with clearly hypointense nodule(s) (*P*=0.002; Figure 5A). The patients with mixed hypointense nodule(s) showed a tendency of worse disease-free survival compared with those of patients with clearly hypointense nodule(s), but the difference did not reach statistical significance (P=0.068; Figure 5B). In regard with OATP1B3 expression, no significant difference was found between the OATP1B3 positive group and negative group in both overall survival and disease-free survival (*P*=0.411, 0.541, respectively; Supplementary Figure 3).

## DISCUSSION

Gadoxetic acid is a potent hepatocyte-specific MRI contrast agent that selectively and robustly enhances the normal liver tissue, and has a proven role to improve the delineation and detection of space occupying lesions [13]. This finding has led to a widespread stereotype among clinicians and radiologists that liver metastases will show minimal or no enhancement on gadoxetic acid-enhanced MRIs. However, recent publications have reported that tumor enhancement quite frequently occurs in CRLMs and breast cancer liver metastases on gadoxetic acid-enhanced MRIs, the underlying cause of which was vaguely assumed to be the result of passive diffusion of the contrast agent into the interstitial space [6, 15]. Along similar lines, we observed mixed hypointensity on MRIs in up to 68% (n=65/96) of CRLMs. However, our data showed an inverse correlation with fibrosis grade in the non-chemotherapy group and no identifiable correlation in the chemotherapy group, suggesting that fibrosis may not be as important a factor as previously assumed. Instead, we found that the mixed hypointense CRLM was associated with positive OATP1B3 immunoreactivity of tumor cells in patients who did not receive chemotherapy.

OATP1B3 is a highly active membrane carrier protein that is responsible for gadoxetic acid transport into hepatocytes. In addition, a subpopulation of hepatocellular carcinomas and adenomas are also known to express OATP1B3, resulting in significant tumor enhancement on gadoxetic acid-enhanced MRIs [16, 17]. The native OATP1B3 protein expressed in normal hepatocytes and these tumors of hepatocyte origin typically shows membranous distribution [18, 19]. Meanwhile, accumulating evidence has indicated that aberrant OATP1B3 expression occurs in a significant proportion of tumors of non-hepatocyte origin, such as colon cancer, breast cancer, pancreatic cancer, and prostate cancer [10–14, 18, 20]. The aberrant OATP1B3 protein produced in these tumors seems to differ from that of native OATP1B3 protein in that the aberrant version typically contains mutations and has a cytoplasmic rather than membranous distribution, implying that the aberrant copies of OATP1B3 might be functionally defective.

To the best of our knowledge, no study has elucidated whether aberrant OATP1B3 proteins (which are potentially functionally defective) expressed in tumors of non-hepatocyte origin have an influence on the enhancement profile of a gadoxetic acid-enhanced MRI. Similar to previous reports that described the cytoplasmic distribution of aberrant OATP1B3 protein [21, 22], all of the CRLMs with positive OATP1B3 expression in our study showed cytoplasmic not membranous distribution of the transporter (Figure 1C). In addition, the aberrant expression of OATP1B3 was associated with mixed hypointensity of CRLMs on gadoxetic acid-enhanced MRIs in the non-chemotherapy group, which we interpret to be the result of preserved (but probably attenuated) transport function of this carrier protein. Our conclusion is compatible with the experimental data based on a colon cancer cell line, which demonstrated that aberrant OATP1B3 protein was capable of transporting several substrates - including gadoxetic acid, despite its cytoplasmic distribution [22].

By contrast, we have failed to identify any association in the chemotherapy group between OATP1B3 expression and the enhancement pattern on MRI. Theoretically, chemotherapy can impair OATP1B3 function by causing cellular damage [23] and by aggravating fibrosis and necrosis. Therefore, we speculated that chemotherapy may consequently provoke passive retention of gadoxetic acid within the extracellular space irrespective of OATP1B3 function. Collectively, the mixed effect of the passive retention of a contrast agent in the stroma and the variable degree of OATP1B3 carrier function (especially after chemotherapy) probably results in variable signal intensity in the HBP on gadoxetic acid-enhanced MRIs [24, 25].

Our results demonstrated that mixed hypointense CRLMs are associated with a worse prognosis compared to that of clearly hypointense lesions. Interestingly, several studies have reported that aberrant OATP1B3 expression is associated with a poor prognosis. Lee *et al.* demonstrated that aberrant OATP1B3 overexpression in a colon cancer cell line interferes with the p53 signaling pathway, providing experimental evidence that OATP1B3 overexpression is associated with reduced apoptosis and the survival advantage of cancer cells [13]. Similarly, colon cancer patients with OATP1B3 overexpression revealed by immunohistochemistry was reported to display a worse progression-free survival rate when compared with patients with scanty or negative OATP1B3 expression [26]. However, in our study we could not detect significant influence of OATP1B3 expression on overall survival and disease free survival. Collectively, we believe that mixed hypointensity of a CRLM is a feature that can be applied as a potential imaging biomarker to screen for high-risk patients, although we do not exactly understand the underlying mechanism that was responsible for the difference in clinical outcome.

There are several limitations to this study. First, there might be selection bias because we only enrolled surgically resected CRLMs larger than 2 cm. Also, the lack of inclusion for patients who received chemotherapy in combination with non-surgical local treatments such as radiofrequency ablation or those who underwent non-curative resection limits the application of our conclusion to certain clinical settings. Second, the retrospective nature of the study prevented us from standardizing the treatment protocol and imaging parameters and matching the histopathologic examination and MR imaging completely slice-by-slice. The variability in chemotherapy regimen could have had some influence on the HBP enhancement pattern and clinical outcome. Third, we were not able to provide experimental evidence regarding the carrier function of aberrant OATP1B3 proteins in terms of their capacity for gadoxetic acid transport. Fourth, the state of OATP1B3 expression did not show direct influence on clinical outcome. Therefore, we were not able to explain the underlying mechanism why mixed hypointense CRLM lesions were associated with worse prognosis. Fifth, aberrant OATP1B3 expression was observed in both chemotherapy and non-chemotherapy groups but was significantly associated with the mixed hypointensity on gadoxetic acid-enhanced MRI only in the non-chemotherapy group. We were not able to clarify the reason why OATP1B3 expression did not seem to affect the MRI enhancement profile in the chemotherapy group. To resolve the latter two limitations, we believe that a genetic analysis including, but not limited to, DNA and/or mRNA sequencing of the aberrant copies of *OATP1B3* is required, but this analysis is not feasible using formalin-fixed, paraffin-embedded samples.

In conclusion, a considerable portion of CRLMs showed mixed hypointensity on gadoxetic acid-enhanced MRI that seems to be a phenomenon poorly linked with fibrosis, but instead can be accounted for by the aberrant tumor expression of OATP1B3 at least in the non-chemotherapy group. Mixed hypointense CRLMs had potential association with a worse prognosis, and, therefore, we believe that this enhancement pattern can be applied as a potential imaging biomarker.

## MATERIALS AND METHODS

This retrospective study was approved by the institutional review board and a waiver for informed consent was obtained.

### Patients

We analyzed 208 consecutive CRLM patients who underwent a curative surgical resection at our hospital from January 2010 to June 2012. We excluded patients with 1) a history of previous local treatment such as radiofrequency ablation therapy or a transcatheter arterial chemoembolization (TACE; n=27), 2) patients who lacked pre-operative gadoxetic acid–enhanced MRI (n=13), 3) patients who had a poor quality pre-operative gadoxetic acid-enhanced MRI (n=2), and 4) patients who had nodules in which the maximum diameter of the lesion was <2 cm on the MRI to minimize the error in the ROI measurement and visual assessment (n=70; Supplementary Figure 1). Therefore, our final study population consisted of 96 histologically proven CRLM patients [60 men and 36 women; median age = 58 years (30-81 years)]. 96 CRLM patients had total of 129 nodules (nodules per patient, 1.34; range, 1-6). We selected the largest nodule for analysis in 20 patients with multiple CRLMs because CRLMs in the same patient showed similar characteristics. Finally our study group consisted of 96 nodules in the 96 histologically proven CRLM patients. Among the 96 patients, 55 (57%) patients did not have a history of chemotherapy, and 41 patients (43%) had received chemotherapy at the time when the MRI was performed. The chemotherapy regimens used in the chemotherapy group (n=41) were as follows: FOLFOX (folinic acid, 5-fluorouracil, and oxaliplatin; n=24, 59%), FOLFOX+bevacizumab (n=8, 20%), FOLFOX+cetuximab (n=4, 10%), FOLFIRI (folinic acid, 5-fluorouracil, and irinotecan; n=4, 10%), SOX (S-1 and oxaliplatin; n=1, 2%). We searched the medical records for variables, including preoperative laboratory results, preoperative MR images, surgical records, pathologic reports, the post-operative course, and the long-term follow-up course.

### Imaging techniques

All patients underwent a pre-operative liver MRI. The mean time interval between preoperative gadoxetic acid-enhanced MRI and surgery was 35 days (range: 1-171days). These studies were performed on 3.0 T MR scanners for 96 patients (Magnetom Trio Tim; Siemens Medical Solutions, Erlangen, Germany [n=66] or Achieva; Philips Medical Systems, Best, the Netherlands [n=30], with a body phased-array coil).

Contrast-enhanced dynamic MRI was performed after a rapid bolus injection of gadoxetic acid disodium (Primovist, Bayer Schering; 0.025 mmol/kg), followed by a saline flush of 15–20 ml with an injection rate of 2 mL/sec. A three-dimensional (3D) T1-weighted spoiled GRE sequence with chemically selective fat suppression (2.54–3.03 ms/0.9–1.4 ms/13°/256×192/2 mm/0 mm) was performed at 25–30 seconds (arterial phase), 55–70 seconds (portal phase), 100–180 seconds (transitional phase), and 15–20 minutes (hepatobiliary phase). T2-weighted images were obtained by multi-shot and single-shot turbo spin echo sequences using a navigator-triggered technique, with a section thickness, gap, repetition time, and echo time of 5–7 mm, 1 mm, 1589–3250 msec, and 70–96 msec, respectively.

### Image analysis

#### Qualitative analysis

The HBP phases of liver MR imaging were reviewed in consensus by a radiologist (J.Y.C.) with 16 years of experience in liver MRI and by a radiology resident (S.H.P.). These radiologists were blinded to all clinical and pathology data. We evaluated the SI of the largest CRLM relative to the background liver parenchyma. CRLMs were defined as clearly hypointense nodules if the signal intensity of the nodule showed a homogenous low signal intensity similar to that of the hepatic segment of inferior vena cava. A mixed hypointense nodule was defined as a lesion (at least partly) showing a signal intensity higher than that of the inferior vena cava.

### Quantitative analysis

Quantitative parameters were calculated by drawing the region of interest (ROI) on the HBP image displayed by the PACS system (GE Medical Systems, Milwaukee, WI, USA). One investigator (S.H.P.) drew the maximum ROI at the largest diameter of the tumor and similar sized ROI in the adjacent homogenous liver parenchyma, avoiding vessels and the bile duct. Four parameters were calculated by the following equations. (1) The relative intensity ratio on the HBP image (RIR_post_) = SI of the nodule on the HBP image/SI of the liver parenchyma on the HBP image. (2) The relative intensity ratio on the precontrast image (RIR_pre_) = SI of the nodule on the precontrast image/SI of the liver parenchyma on the precontrast image. (3) The relative enhancement ratio (RER) = RIR_post_/RIR_pre_. (4) The contrast to noise ratio (CNR) = (SI of the nodule on the HBP image – SI of the liver parenchyma on the HBP image)/standard deviation (SD) of the liver parenchyma on the HBP image.

### Histopathology analysis

Formalin-fixed, paraffin-embedded specimens from the nodules examined on MRI were selected for histopathologic analyses. Consecutive sections were obtained for H&E staining and immunohistochemistry. H&E stained specimens were reviewed by a pathologist (E.K.K.) for a tumor component analysis. The pathologist was aware that the specimens were colorectal cancer liver metastatic lesions as well as the location and size of the tumors to match the corresponding imaging but was blinded to all other clinical and MRI data. A semi-quantitative 4-point scoring system was applied to measure fibrosis, necrosis, and cellularity by the following criteria: grade 1 (G1), the area of each component was <25% of the tumor area; grade 2 (G2), 25% to 50% of the tumor area; grade 3 (G3), 50% to 75% of the tumor area, and grade 4 (G4), >75% of the tumor area. In this study, grade 1 was defined as “low-grade” while grades 2-4 were defined as “high-grade”.

Immunohistochemistry was performed using a rabbit polyclonal anti-human OATP1B3 antibody (HPA004943; Sigma–Aldrich, St. Louis, MO, USA) as previously described [13]. Briefly, each section was deparaffinized, rehydrated, and antigen retrieved. The sectioned slides were incubated for 1 hour at room temperature with blocking solution containing 5% goad serum in PBST (0.3% Triton X-100 in phosphate buffered saline). After blocking, the sectioned samples were incubated overnight at 4°C with the primary antibody (rabbit polyclonal anti-human OATP1B3 antibody). After several washes in PBST, the slides were incubated with horseradish peroxidase-conjugated anti-rabbit IgG antibody. The immune reaction was visualized using 3,3’-diaminobenzidine (DAB). The slides were counter-stained with hematoxylin. The specificity of immunohistochemical staining was verified by omitting either the primary or secondary antibody.

The staining intensities were evaluated by a pathologist (E.K.K.) using the following categories: negative, weakly positive, moderately positive, and strongly positive (Supplementary Figure 4). The normal colonic mucosa shows negative OATP1B3 staining and therefore sections scored as weakly positive or higher were defined as OATP1B3-positive [13].

### Statistical analysis

All statistical analyses were performed using IBM SPSS 20.0 software (IBM Corp., Armonk, NY, USA). A Chi-square test was performed to compare the enhancement pattern between the non-chemotherapy group and the chemotherapy group. A univariate binary logistic regression was performed to evaluate the relationship between the histopathologic components and the qualitative enhancement pattern. Thereafter a multivariate binary logistic regression was performed for all variables. By a similar method, univariate and multivariate linear logistic regressions were performed for the quantitative parameters. To compare disease-free survival and overall survival, a Kaplan-Meier survival analysis and a log-rank test were used. Patients lost to follow-up or that died due to unrelated causes were regarded as censored data. A value of *P*<0.05 was considered to be statistically significant.

## SUPPLEMENTARY MATERIALS FIGURES



## Abbreviations

CEA: carcinoembryonic antigen
CNR: contrast to noise ratio
CRC: colorectal cancer
CRLM: colorectal cancer liver metastases
HBP: hepatobiliary phase
LM: liver metastases
MRI: magnetic resonance imaging
OATP1B3: organic anion transporting polypeptide 1B3
RER: relative enhancement ratio
RIR_post_: relative intensity ratio on the HBP image
RIR_pre_: relative intensity ratio on the precontrast image
ROI: region of interest
SI: signal intensity
TACE: transcatheter arterial chemoembolization
