# Supplementary material for: Aberrant expression of OATP1B3 in colorectal cancer liver metastases and its clinical implication on gadoxetic acid-enhanced MRI
Source: Oncotarget. 2017 Aug 16;8(41):71012–23. doi: 10.18632/oncotarget.20295 (PMC5642614; doi:10.18632/oncotarget.20295)
Supplement: Supplementary file 1 [file oncotarget-08-71012-s001.pdf]

Aberrant expression of OATP1B3 in colorectal cancer liver metastases and its clinical implication on gadoxetic acid-enhanced MRI

SUPPLEMENTARY MATERIALS

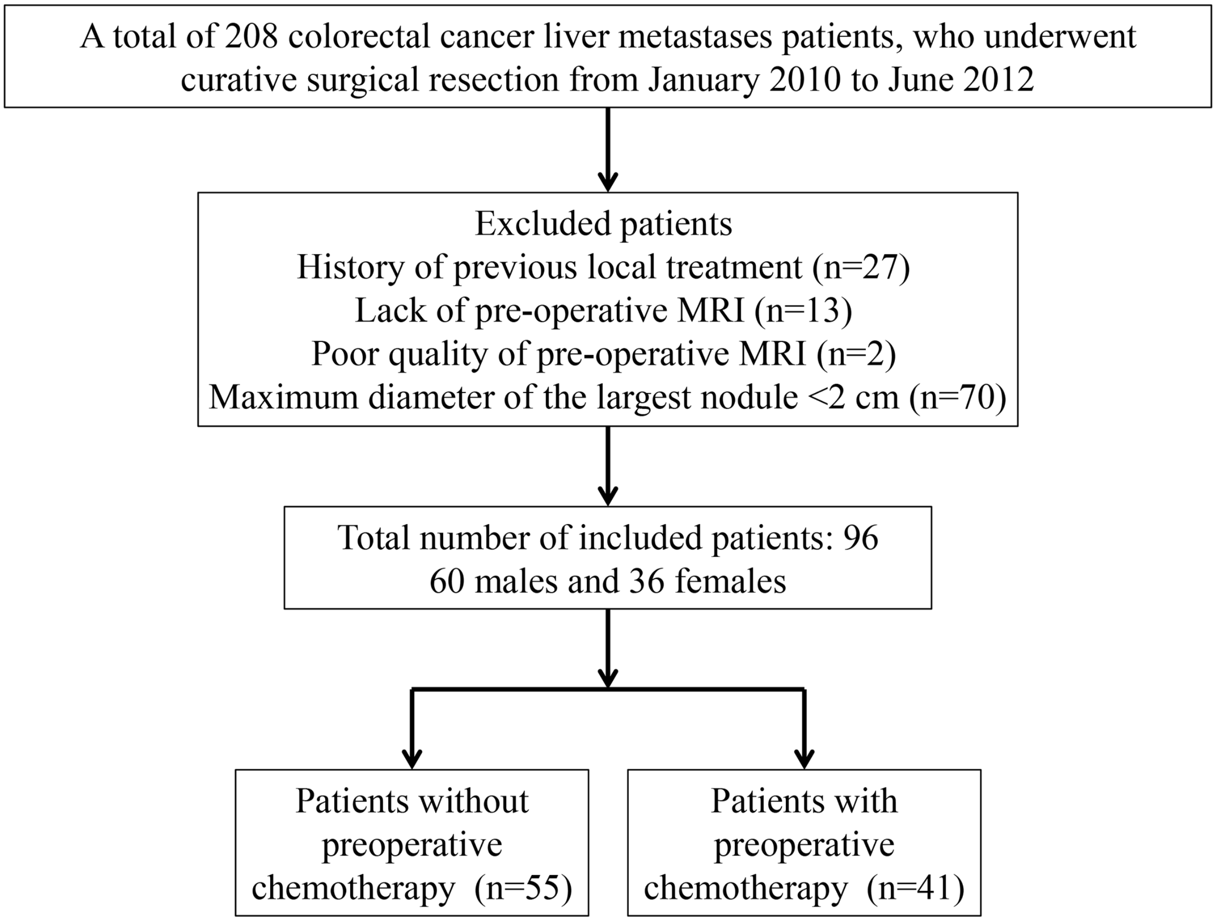

Supplementary Figure 1: Eligibility criteria for the study population.

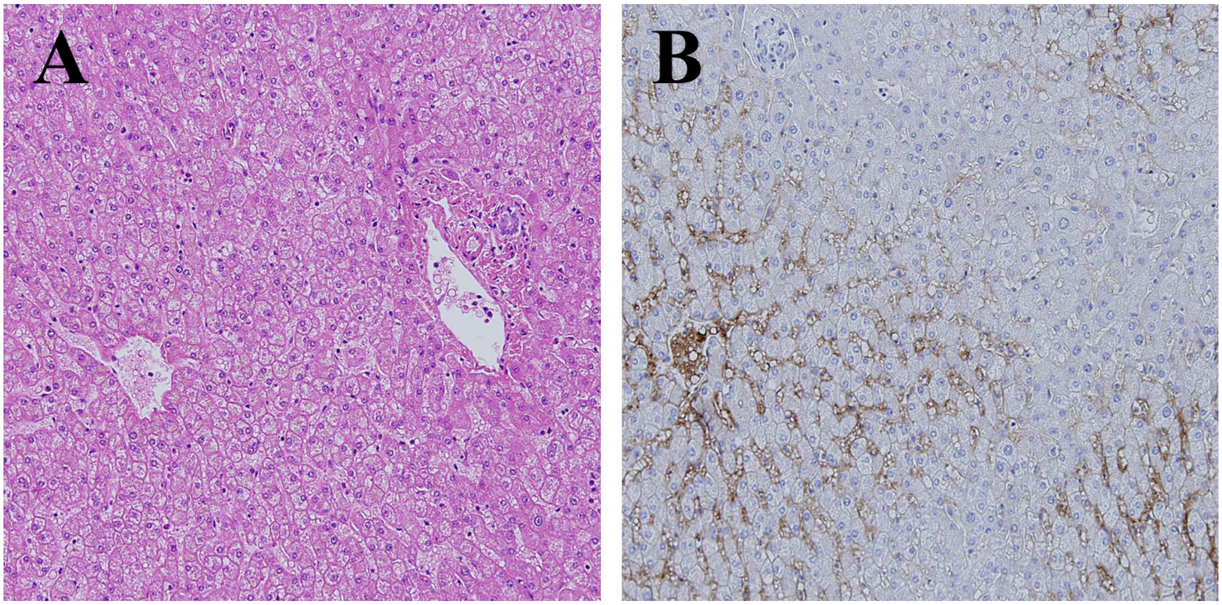

Supplementary Figure 2: Histopathology of a tumor-free liver (uninvolved CRLM) in a 50-year-old woman. **(A)** Hematoxylin and eosin staining shows normal liver architecture. **(B)** The immunohistochemistry (×100) of normal hepatocytes shows intense membranous expression of OATP1B3.

A

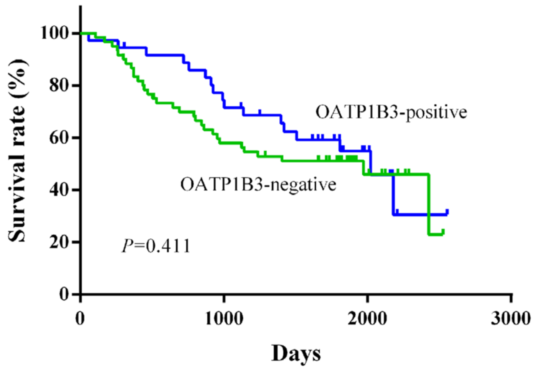

Follow-up time (Days)	0	500	1000	1500	2000	2500
OATP1B3-negative						
Events	0	14	25	29	30	31
Number at risk	60	46	34	29	9	1
OATP1B3-positive						
Events	0	3	9	13	15	17
Number at risk	36	32	26	20	9	1

B

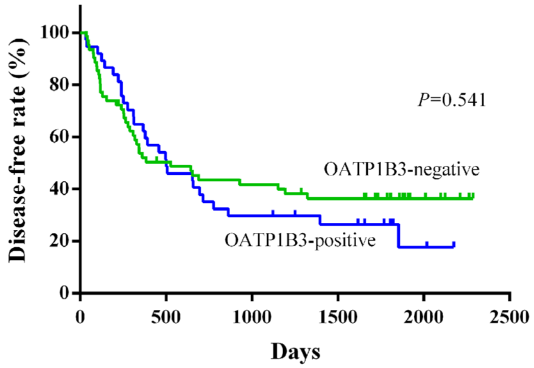

Follow-up time (Days)	0	500	1000	1500	2000	2500
OATP1B3-negative						
Events	0	29	34	37	37	37
Number at risk	60	29	24	20	6	0
OATP1B3-positive						
Events	0	18	25	26	27	27
Number at risk	36	18	11	8	2	0

Supplementary Figure 3: Kaplan-Meier survival curves in CRLM patients based on the OATP1B3 expression of the tumor.

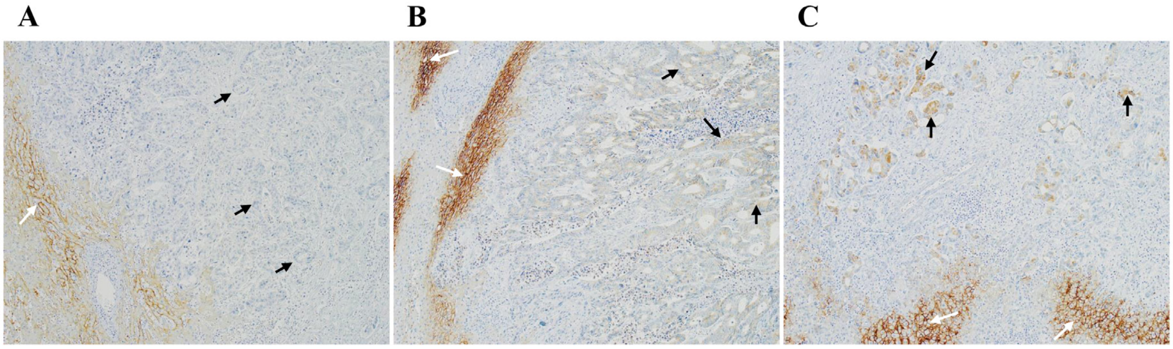

Supplementary Figure 4: Representative microscopic images (×100) of the OATP1B3 immunohistochemistry staining demonstrating. (A) OATP1B3 negative**, (B)** weak positive and **(C)** moderately positive CRLM tumor cells (black arrows). Note that the adjacent tumor free liver showed strong positive, membranous OATP1B3 expression (white arrows) whereas OATP1B3 positive CRLM tumor cells displayed a cystoplasmic distribution of immune reaction (B, C).
